# Supplementary material for: Structural and Genetic Determinants of Convergence in the Drosophila tRNA Structure–Function Map
Source: J Mol Evol. 2021 Feb 2;89(1):103–16. doi: 10.1007/s00239-021-09995-z (PMC7884595; doi:10.1007/s00239-021-09995-z)
Supplement: Supplementary file 1 — Supplementary file1 (PDF 378 KB) [file 239_2021_9995_MOESM1_ESM.pdf]

Supplementary Online Materials for Phillips and Ardell "Structural and Genetic Determinants of Convergence in the *Drosophila* tRNA Structure-Function Map"

CONTENTS

1 Code and Data 1

2 Substitutions in Ion-Binding Pocket by Functional Class and Location 1

3 Substitutions in Ion-Binding Pockets by Chromosomal Location 3

4 Transition and Transversion Rates in *Drosophila* tRNA Genes 3

5 Site Divergences with Other Evolutionary Models 3

6 Significance of Paired-Site CIFs in *D. melanogaster* 3

7 Average Frequency and Information of Nucleotides in tRNA genes in *D. melanogaster* 3

1. CODE AND DATA

The full code and data to reproduce all results is available at http://dx.doi.org/10.6084/m9.figshare.12713705.

2. SUBSTITUTIONS IN ION-BINDING POCKET BY FUNCTIONAL CLASS AND LOCATION

Table S1. Sequence patterns, ortholog sets, functional classes and locations (in *D. melanogaster*) contributing to elevated substitution rates in an ion-binding pocket. Results are provided for the largest subset of data partitions (X indicates the species is excluded from that partition). Species bit-string corresponds in order to: *D. melanogaster, D. simulans, D. sechellia, D. yakuba, D. erecta, D.ananassae, D pseudoobscura, D. persimilis, D. willistoni, D. mojavensis, D. virilis, D. grimshawi*. Functional classes use IUPAC amino acid one-letter abbreviations for elongator tRNAs, “X" for initiator tRNAs, “U" for selenocysteine tRNAs.

Species Bit-String	Coordinate(s)	Ortholog Set (Class Anticodon):Sequence	Location in *D. melanogaster*
111111110111	16:17	72 (U TCA):TGGGGGGG0GGG:<br>-TTTTTTTTTT	2R:c(7245069..7245155)
111111110111	16:60	2 (R TCT):TTTTTCCC0TCC:<br>TTTTTCCCTCC	2L:1965426..1965498
		92 (L AAG):CCCCCCCC0CCT	2R:c(10871832..10871913)
		112(I AAT):TTTTTTTT0CCC	2R:15603070..15603143
		114(X CAT):TTTTTTCT0TTT	2R:15613410..15613481
111111110111	16		

Table S1 – continued from previous page

Species Bit-String	Coordinate(s)	Ortholog Set (Class Anticodon):Sequence	Location in *D. melanogaster*
		136(S TGA):TTTTTCTT0TTT	2R:c(18959542..18959623)
		137(S TGA):TTTTTCTT0TTT	2R:18960104..18960185
		188(P CGG):AAAAAAAA0GGG	3L:18611490..18611561
		192(R TCG):AAAAAAAA0TTT	3R:1213950..1214022
		226(V AAC):TTTTTTCT0TTT	3R:12147412..12147484
		251(V CAC):CCCCCCTC0TCC	3R:15615844..15615916
		285(S AGA):CCCCCCCC0ACC	X:c(13919142..13919223)
111111110111	17	46 (N GTT):TTTTTCTT0CCT	2R:c(2040108..2040181)
		77 (M CAT):TTTTTTTT0ATT	2R:c(7548068..7548140)
111111110111	17	102(F GAA):T-TTTTTT0TTT	2R:c(13492040..13492112)
		138(N GTT):TTTTTCTT0CCT	2R:20261586..20261659
		186(M CAT):TTTTTTTT0AAA	3L:16343017..16343089
		205(N GTT):TTTTTCTT0CCT	3R:c(3965255..3965328)
111111110111	60	231(A AGC):GGGGGAGG0GGG	3R:13445460..13445532
		240(A AGC):GGGGGAGG0GGG	3R:c(13471029..13471101)
		245(A AGC):GGGAGAGG0AGG	3R:c(13484103..13484175)
		246(A AGC):GGGGGAGG0AGG	3R:13493909..13493981
		252(A AGC):GGGGGAGG0GGG	3R:15616694..15616766
11111111011X	16	260(S GCT):TTTTTATT0AAX	3R:c(18222902..18222983)
11111111011X	60	221(T TGT):CCCCCCTT0TCX	3R:c(8148285..8148356)
		242(A AGC):GGGGGAGG0GGX	3R:13472090..13472162
1X111111011X	16	258(S GCT):AXAAATAA0TTX	3R:18222250..18222331
		259(S GCT):AXAAAAAA0TTX	3R:c(18222521..18222602)
111111110XXX	16	83 (I AAT):TCCCTTCC0XXX	2R:c(9317787..9317860)
		85 (I AAT):TTTCTTCC0XXX	2R:9318489..9318562
		272(Q TTG):CCCTTTTT0XXX	X:c(3321485..3321556)
111111110XXX	17	193(M CAT):TTTTTAAA0XXX	3R:c(2321761..2321833)
111111110XXX	60	234(A AGC):GGGGGAGG0XXX	3R:13448268..13448340
		238(A AGC):GGGGGAGG0XXX	3R:c(13456764..13456836)
		244(A AGC):AGGGGAGG0XXX	3R:13482867..13482939
11XX111X0111	16	287(S AGA):TTXXTTCX0CCC	X:13964674..13964755
11X1111X01X1	16	292(P CGG):AAXAAGTX0TXT	X:18459797..18459868
1111X1110XXX	17	61 (N GTT):TTTTXCTT0XXX	2R:c(2077634..2077707)
1XXX111X0111	60	166(A AGC):GXXXGAGX0GGG	3L:c(8021646..8021718)
111111X10XXX	16	273(Q CTG):TTTTTTXC0XXX	X:3713732..3713803
		294(I AAT):TTTTTTXC0XXX	X:c(21102352..21102426)
1XX1111X01X1	16	220(T TGT):TXXTTTTX0TXA	3R:8032297..8032368

Table S1 – continued from previous page

Species Bit-String	Coordinate(s)	Ortholog Set (Class Anticodon):Sequence	Location in *D. melanogaster*
1X1111110XXX	16	274(P CGG):GXGAAGTT0XXX	X:3721655..3721726
		289(R TCG):TXTTTTCC0XXX	X:c(13997752..13997824)
1X1111110XXX	17	47 (N GTT):TXTTTCTT0XXX	2R:2040691..2040764

3. SUBSTITUTIONS IN ION-BINDING POCKETS BY CHROMOSOMAL LOCATION

4. TRANSITION AND TRANSVERSION RATES IN *DROSOPHILA* TRNA GENES

5. SITE DIVERGENCES WITH OTHER EVOLUTIONARY MODELS

We ran analogous site divergence calculations using the HKY model (Hasegawa, M., H. Kishino, and T. Yano (1985). Dating of the human-ape splitting by a molecular clock of mitochondrial DNA. *Journal of Molecular Evolution* 22(2), 160–174) and the HKY+Γ model. We estimated substitution rates with MrBayes 3.2.1 (Huelsenbeck and Ronquist 2001; Ronquist et al. 2012) using the fixed known species tree (Drosophila 12 Genomes Consortium 2007). For all runs we constrained change to the tree topology by setting rates of stochastic TBR and branch multipliers to zero probability. All Bayesian analyses were run with two simultaneous chains for 4×10^6 iterations, monitoring convergence of split frequency standard deviations and saved parameters every 500 iterations.

6. SIGNIFICANCE OF PAIRED-SITE CIFS IN *D. MELANOGASTER*

7. AVERAGE FREQUENCY AND INFORMATION OF NUCLEOTIDES IN TRNA GENES IN *D. MELANOGASTER*

Table S2. Sequence changes in ion-binding pocket sites by chromosome (Muller elements A – E), when location data was available for *D. melanogaster*. The X chromosome (Muller element A) has a larger fraction of change compared to other chromosome arms.

Chromosome Location	Ortholog Sets with Changes in Sites 15, 18, 19, 20, or 59 (% of total)	Ortholog Sets with Changes in Sites 16, 17 or 60 (% of total)
2L	5 (0.119)	4 (0.095)
2R	10 (0.096)	23 (0.221)
3L	6 (0.113)	7 (0.132)
3R	6 (0.073)	21 (0.256)
X	9 (0.273)	11 (0.333)
Not Available	14 (0.030)	18 (0.039)

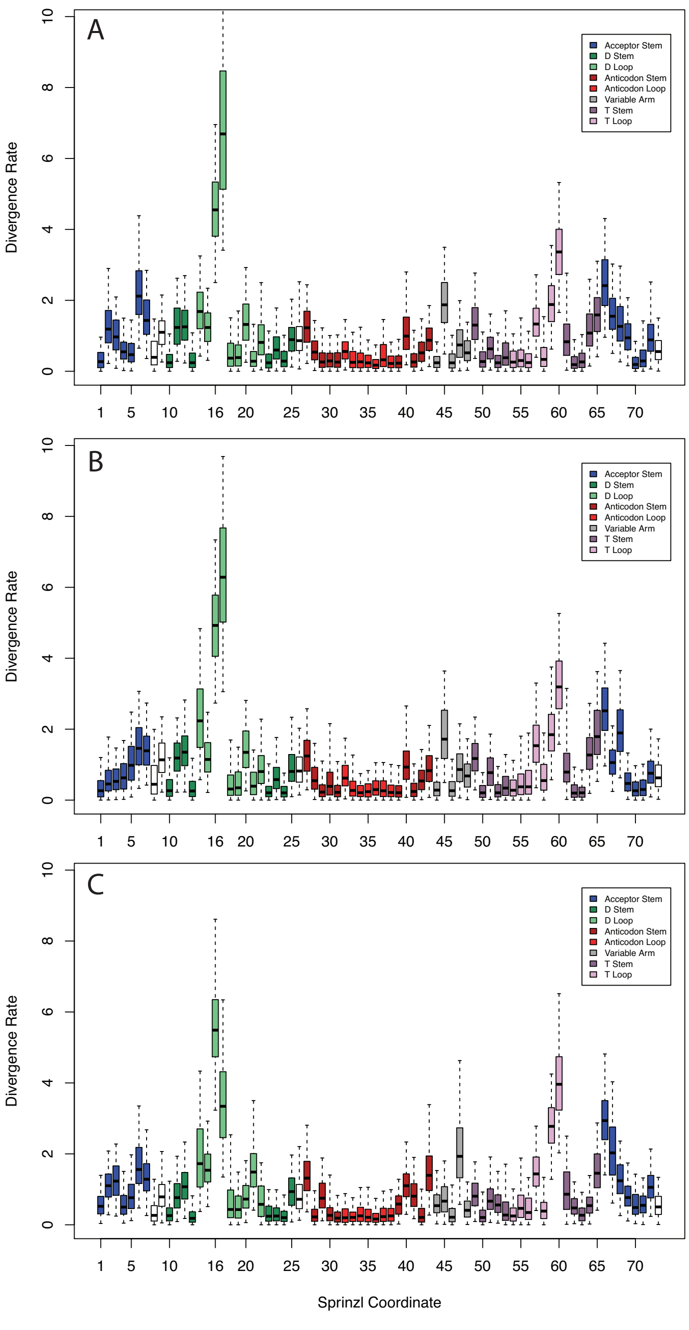

Fig. S1. Site divergence rates with a HKY evolutionary model. (A) the set with the largest number of species (111111110111, missing *D. willistoni*); 84 orthologous sets); (B) the set with the second largest tree length (110011100111, second only to the set with largest number of species, set contains *D. melanogaster, D. simulans, D. erecta, D.ananassae, D pseudoobscura, D. mojavensis, D. virilis, D. grimshawi*); 86 orthologous sets; and (C) the set with the largest number of included ortholog sets (101111110000, *D. melanogaster, D. sechellia, D. yakuba, D. erecta, D.ananassae, D. pseudoobscura, D. persimilis*); 155 ortholog sets

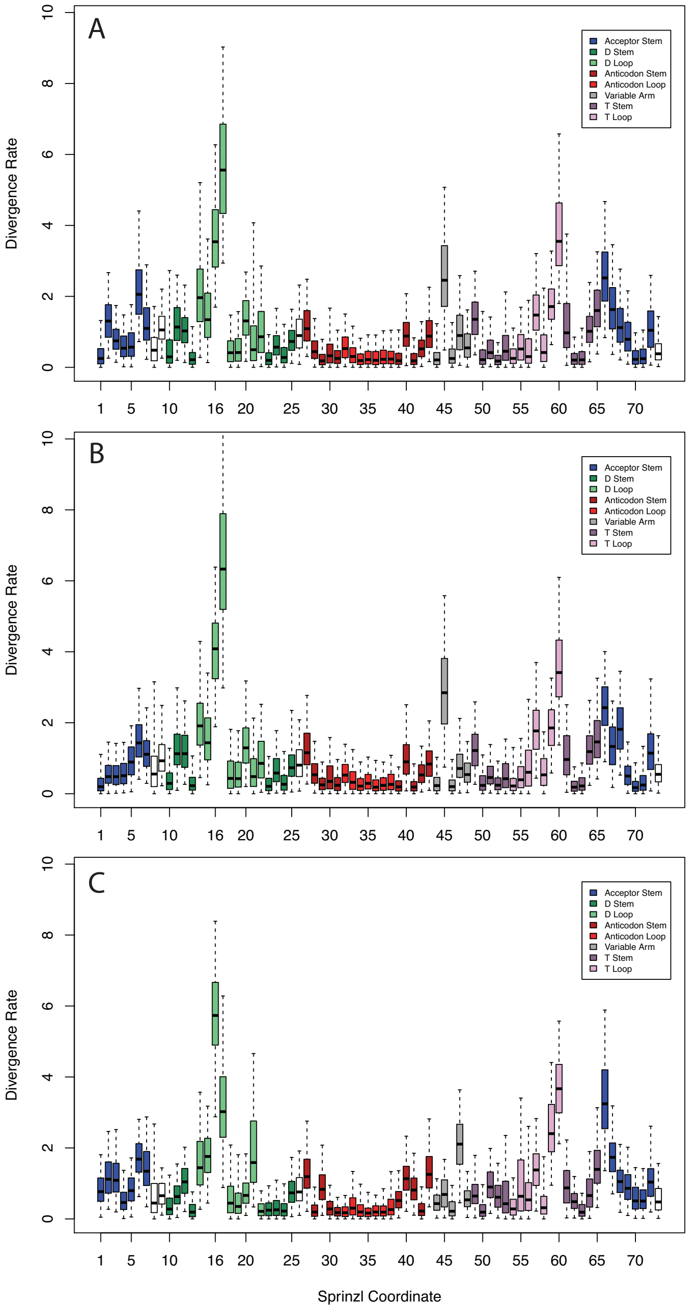

Fig. S2. Site divergence rates for three Mr.Bayes analyses show confirmatory results to the GTR model when simulations are run with a HKY+γ evolutionary model. Sites show similar patterns to the structural divergence rates in the GTR model data.. (A) the set with the largest number of species (111111110111, missing *D. willistoni*); 84 orthologous sets); (B) the set with the second largest tree length (110011100111, second only to the set with largest number of species, set contains *D. melanogaster, D. simulans, D. erecta, D.ananassae, D pseudoobscura, D. mojavensis, D. virilis, D. grimshawi*); 86 orthologous sets; and (C) the set with the largest number of included ortholog sets (101111110000, *D. melanogaster, D. sechellia, D. yakuba, D. erecta, D.ananassae, D. pseudoobscura, D. persimilis*); 155 ortholog sets

Table S3. Median and Credible Intervals of Transition and Transversion Rates in *Drosophila* tRNA Genes

	**101111110000**	**110011100111**	**111111110111**
**C↔T**	0.2726 (0.1725, 0.3756)	0.2491 (0.1402, 0.3657)	0.2662 (0.1979, 0.3875)
**A↔G**	0.4828 (0.3557, 0.6201)	0.4947 (0.3325, 0.6455)	0.4889 (0.4031, 0.6464)
**A↔C**	0.0456 (0.0053, 0.0942)	0.1045 (0.0353, 0.1951)	0.1047 (0.0510, 0.1857)
**G↔T**	0.0676 (0.0250, 0.1140)	0.0508 (0.0147, 0.0914)	0.0472 (0.0224, 0.0873)
**A↔T**	0.0600 (0.0191, 0.1078)	0.0427 (0.0048, 0.0882)	0.0426 (0.0158, 0.0878)
**C↔G**	0.0714 (0.0297, 0.1161)	0.0583 (0.0192, 0.1036)	0.0504 (0.0286, 0.0912)

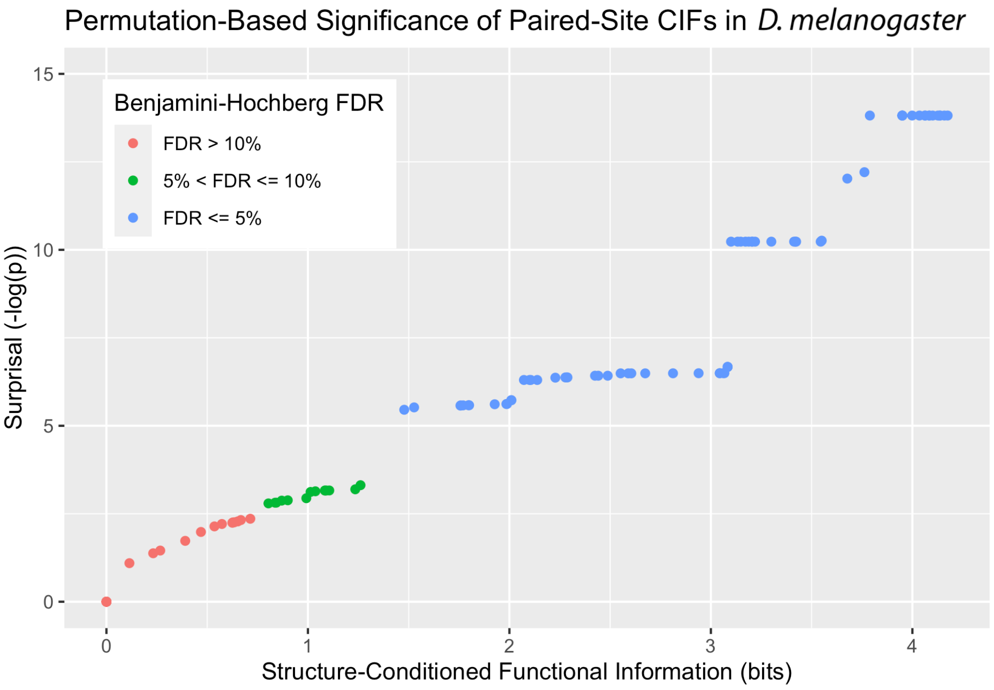

Fig. S3. The Surprisal of paired-site tRNA CIFs in *D. melanogaster* as a function of the total structure-conditioned functional information (stack-heights) of CIFs, and colored by computed Benjamini-Hochberg FDRs, as computed by tSFM v1.0.

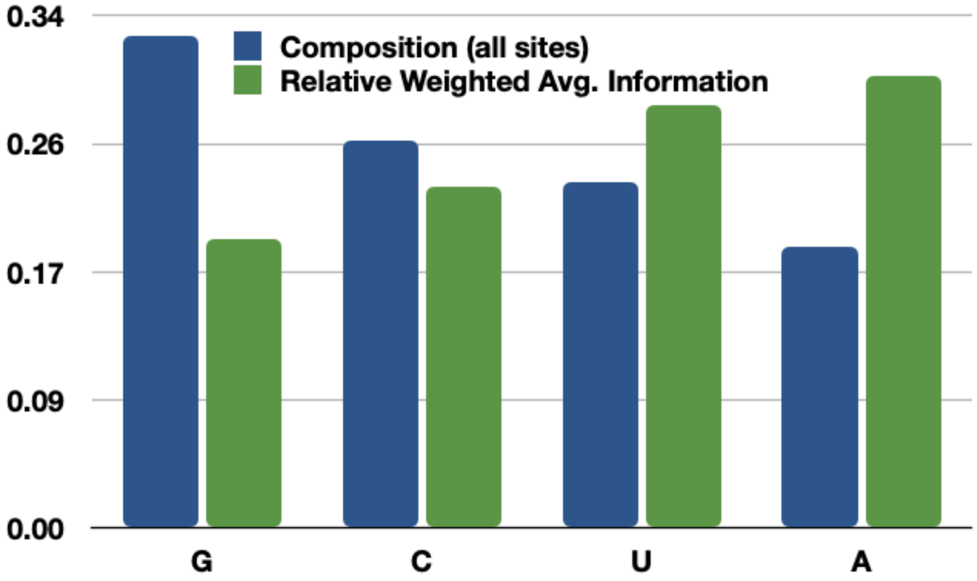

Fig. S4. Average composition and relative average structure-conditioned functional information of the four RNA nucleotides in 288 *Drosophila* tRNA genes with COVE bit-scores $\geq$ 50 bits.
